# A Quack Advertisement

**Published:** 1859-10

**Authors:** 


### TAKE NOTICE.

The author would with pleasure, Inform his friends & The public in general that he has located at concord Station Concord Erie Co penn On the Sunbury & Erie, R. R.

Where he will pay strict attention to Diseases in general, and most strict attention to Female & cronic disease's such as Prolapsis, Flouralbus Barrenness Menstruation,s & General nervous debilityes of the Female system to geather with cronic Rheumatis Piles Kidney & Liver Old & Ulcerated Sores Saltrhume Irresypulous Feaver Sores White swellings &c &c cansors that are not to far advanced easily removed

We go to the wilds of the East & the west the River & Mountain for chois remmidies which God planted for our bennifit

To be consulted at my office on wednesday Pm, & sunday invariably

July, 4, 1859. DR ———

We have received the above notice from an esteemed correspondent, with the request, with which we cheerfully comply, that we publish it *verbatim et literatim.* Our correspondent is justly indignant at the assurance of such "ignoramuses," and quite properly lays a great deal of the annoyance which they occasion to the regular practitioner, to the door of the respectable and intelligent people who persist in giving them encouragement. He makes a remark, which every practicing physician knows to be perfectly true, viz., "plenty of people will leave their own intelligent, educated physicians at home, and go a long journey to procure the advice of such a monstrous ass." Were it not for those who should know better, whom we should expect to set a better example, the bounds of quackery would be much curtailed. We think that we are borne out by observation, when we state that the lower classes of Irish and Germans are not so apt to trust in charlatans as those in the higher walks of life; we are, indeed, frequently called upon to prescribe for servants in families who employ homœopathists, who cannot be persuaded to trust to the family physician. It is a notorious fact, also, and one which has been commented upon by the medical journals, that clergymen are extremely prone to quacks. This is a blow at the dignity of our profession to which we should not tamely submit. Clergymen always receive their medical services gratuitously, and frequently when they are perfectly able to pay, and the physician struggling for very existence. Nevertheless, we have no objection to make to such a custom, provided we are treated, in return, with the courtesy and respect which is our right. But when we see every quack remedy followed by a long line of testimonials from the clergy; when, as a rule, and we think that we are not speaking too positively on this point, clergymen give their countenance to charlatans oftener than to the regular profession; we think that it is time for us to make exceptions to the rule of rendering them our services gratuitously.

In whose keeping is the honor and standing of our profession? Certainly not in the keeping of those from whom we have a right to expect support. It is only a few among the intelligent men of a community who seem to appreciate what is due to a faithful and conscientious physician. Let us look now nearer home. In scrutinizing our own conduct, cannot we discover some fault? Before we can expect to bring society to a sense of what is due to us as a profession, we must do all we can for ourselves. This we must do by pursuing a straightforward, uncompromising course in regard to irregulars, using the term irregular in its broadest sense. We have no right, in justice to the profession, to consult with irregulars, to countenance them professionally in any way, or even to tolerate them socially. Let us respect ourselves in this regard at least! When one of our own number lapses from the path, cut him off; and at least give no encouragement to quacks of ever being tolerated by the regular profession! This is the course which has been advocated by this Journal now for nearly fifteen years. No respectable journal has been able to stand with any other policy. As for ours, it was started with these principles, and will die with them.

Our remarks have been more extended than we designed, for we warmed with the subject, well worn though it be; but before closing, we wish to suggest another point in which we think we lack a little dignity, through our own fault. We are not every one's servant, and in our professional relations with society, should not submit to slights, inattention, and want of consideration, even if we receive them at the hands of the wealthy and influential. The idea that a wealthy ignoramus who has happened to become rich, confers a favor upon an educated physician when he requests his services, is preposterous; the favor and the condescension, if there be any, is on the other side. When we respect ourselves more in this particular, we will be more respected by others. A moneyed dunce has an innate veneration for mind and attainment, though he sometimes succeeds in concealing it. He wishes to be respected for his wealth, which has been the object of his life. We should equally insist upon the respect due to our attainments, and to our noble profession.

